# Systems-Level Plasma Biomarkers for Tissue Health: Integrating Protein Networks with RAG Biological Insights

**DOI:** 10.1093/geroni/igaf122.2547

**Published:** 2025-12-31

**Authors:** Qiongrong Huang, John Earls, Noa Rappaport, Lance Pflieger, Kengo Watanabe, Nathan Price

**Affiliations:** The Buck Institute, Navato, California, United States; The Buck Institute, Navato, California, United States; Institute for Systems Biology, Buck Institute, Phenome Health, Seattle, Washington, United States; Phenome Health, Seattle, Washington, United States; Tokushima University Graduate School of Biomedical Sciences, Tokushima, Tokushima, Japan; The Buck Institute, Navato, California, United States

## Abstract

Plasma proteomics offers a promising avenue to infer tissue-specific biological processes; however, the regulatory relationships between plasma and tissues remain unclear. Using paired plasma-tissue proteomic data from 172 samples spanning muscle, ovary, and breast tissues, we we analyzed 5,415 proteins and employed correlation analyses and Lasso regression to identify plasma protein signatures predictive of tissue protein levels. To validate and contextualize these findings, we integrated a retrieval-augmented generation (RAG) model powered by large language models (LLMs) to enrich our understanding of systemic protein interactions. We uncovered robust cross-tissue regulatory protein modules independent of age and sex, primarily mediating immune system regulation and steroid hormone. Sex- and age-dependent regulatory modules were identified using weighted gene co-expression network analysis (WGCNA), revealing distinct aging effects: in males, aging impacted modules related to muscular and reproductive system development, angiogenesis regulation, and non-canonical Wnt signaling (Spearman’s ρ = 0.66, p < 2.2 × 10^⁻16^, whereas in females, aging primarily affected cell-cell interactions, angiogenesis, extracellular matrix organization, and nervous system development (Spearman’s ρ = 0.34, p < 2.2 × 10^⁻16^). Plasma-tissue modules exhibited functional parallels with minimal protein overlap, suggesting regulatory mediation via distinct proteins. Using Differential Rank Conservation (DIRAC), we further identified key plasma-tissue interactions associated with longevity. These findings provide a systems-level perspective on the plasma proteome as a noninvasive window into tissue biology, with implications for aging research.

